# *You have to be twice as good and work twice as hard*: a mixed-methods study of perceptions of sexual harassment, assault and women's leadership among female faculty at a research university in the USA

**DOI:** 10.1017/gheg.2019.5

**Published:** 2019-09-05

**Authors:** Dabney P. Evans, Jessica M. Sales, Kathleen H. Krause, Carlos del Rio

**Affiliations:** 1 Hubert Department of Global Health, Rollins School of Public Health, Emory University, 1518 Clifton Road, NE, Atlanta, GA, USA; 2 Department of Behavioral Sciences and Health Education, Rollins School of Public Health, Emory University, Atlanta, GA, USA; 3 Department of Medicine, Emory University School of Medicine, Atlanta, GA, USA

**Keywords:** Gender, leadership, medicine, public health

## Abstract

**Introduction:**

The purpose of this study was to examine the perceptions of institutional policies and practices for the prevention of and response to gender inequities as experienced by female faculty working in the health sciences at a US research university.

**Methods:**

Data from the institution's Faculty Campus Climate Survey (*n* = 260 female faculty) were coupled with qualitative interviews (*n* = 14) of females in leadership positions, exploring campus climate, and institutional policies and practices aimed at advancing women.

**Results:**

Two-thirds (59%) of the female faculty respondents indicated witnessing sexual harassment and 28% reported experiencing sexual harassment. Several organizational themes emerged to address this problem: culture, including cultural change, transparency, and accountability.

**Conclusions:**

The findings reveal the ways in which university culture mimics the larger societal context. At the same time, the distinct culture of higher education processes for recruitment, career advancement – specifically tenure and promotion – are identified as important factors that require modifications in support of reductions in gender inequalities.

## Introduction

Gender equity and related concerns of sexual assault and harassment have received notable attention in scholarship, policy, and most recently, public discourse [1]. The #MeToo Movement has produced a cascade of allegations against men in positions of power engaged in sexual misconduct [2–5]. Reports of sexual harassment, assault, and gender discrimination among female academicians have also been surfacing, including in the health sciences [6–8]. The dearth of women in key health leadership positions, including in global health, despite large numbers of women appointed in this domain has also been highlighted [9, 10]. For those women working in these disciplines in the academy, the topic hits close to home [11, 12].

The World Health Organization (WHO) defines sexual violence broadly as ‘any sexual act, attempt to obtain a sexual act, or other act directed against a person's sexuality using coercion, by any person regardless of their relationship to the victim, in any setting’[13]. In the USA, Title VII of the Civil Rights Act prohibits employment discrimination on the basis of sex; Title IX prohibits sex discrimination within educational programs receiving federal financial assistance [14, 15]. Under these laws, university responsibilities for protection against sexual violence and gender discrimination on campus include both preventive and response measures [14, 15]. Campus climate surveys have been touted as best practice for determining the extent of sexual assault, including sexual harassment, on campus and assessing awareness of the issue among students [16–18]. As far as we are aware, few universities have surveyed faculty in this regard.

The National Intimate Partner and Sexual Violence Survey from the US Centers for Disease Control and Prevention (CDC) routinely asks about lifetime prevalence statistics for rape, attempted rape, intimate partner violence, and stalking [19]. Many colleges and universities asked students to report experiences of sexual violence since coming to campus. We asked faculty about any such experience since being appointed in an academic position. In the summer of 2015, Emory University (Atlanta, Georgia, USA) conducted a comprehensive Faculty Campus Climate Survey (FCCS) to capture the experiences of sexual harassment and training in sex discrimination, knowledge of corresponding legislation, and comfort with guiding students and colleagues through the disclosure process [20].

Research universities play a strong role in mentoring future scholars and global leaders in the health sciences. Emory, as a research institution with considerable strengths in the fields of medicine, science, and global health, has the obligation to consider the ways in which its own institutional policies and practices effect the prevention of and response to gender inequities, including sexual violence, among female faculty working in these fields. Utilizing a subset of data from the 2015 FCCS and new qualitative data from Key Informant (KI) interviews, we explore the role of research universities in preventing and responding to gender inequities through the lens of one university experience.

The purpose of this study was to examine the perceptions of institutional policies and practices for the prevention of and response to sexual harassment, assault, and other gender inequities as experienced by female faculty in the health sciences.

## Methods

### Study site

Established in Atlanta, GA in 1919, Emory University is internationally recognized as having excellence in the health sciences, defined for our purposes as medicine, nursing, and public health. The University is composed of nine academic units and coupled with the highest ranked health care system in the state of Georgia [21, 22]. Emory University, including Emory Healthcare, is the second largest employer in Atlanta with over 31 000 employees and over 15 000 students [23].

### Data collection

The Emory University FCCS was designed by a subcommittee of the Emory University Senate Committee for the Prevention of Sexual Violence to gather data similar to Emory's student-focused Campus Climate Survey. The Department of Defense originally developed these measures about sexual harassment, and MIT adapted them to include in their Campus Climate Survey among students. MIT was one of the first universities to conduct and publically share their survey instrument and results. We used these same measures with the idea to include the entire community in the Campus Climate Survey, and in the absence of any guidance about how to include faculty and staff in these surveys.

Survey items assessed the experiences of sexual harassment during tenure at Emory, as well as additional information tailored to faculty perspectives and reflections on their responsibilities under Titles VII and IX. Individual questions were asked about witnessing sexual harassment or experiencing sexual harassment. One question asked – in combination – was about either witnessing or experiencing an inappropriate comment. Because this item is imprecise, we reported the results of this unique item so as not to bias the other exclusive categories.

To complement the survey and further examine institutional structures, a KI interview guide was developed to explore two primary domains: campus climate related to sexual assault and harassment, and institutional policies and practices aimed at reducing gender inequities and advancing women leaders in the health sciences. While not intended to explore individual experiences of sexual assault or harassment, participants were asked to reflect on how these issues have been handled at Emory and whether they had observed changes over time including changes in institutional policy and practice. Probing, follow-up, and interpretive questions were used to further explore the topics that were brought up during the interviews including positive and negative examples of formal policies and informal practice. Both the quantitative survey instrument and KI interview guide are available upon request.

### Participants

All currently employed faculty and staff members were invited to participate in the FCCS via email, having opportunities to complete it online between 5 July and 31 August 2015. Within a week of closing the survey, a reminder email was sent encouraging participation. Out of 11 631 faculty and staff contacted, 2807 faculty and staff (24%) accessed the survey, and 2290 of those (20%) answered at least one question. Of these respondents, 64% identified as women, 34% as men, <2% identified as transgender, ‘other,’ or with a preference not to respond; 596 respondents identified as faculty and 1667 respondents identified as staff. The mean age of respondents was 43.67 years (standard deviation = 12.77 years; range 18–94 years), with 3.5% of the respondents identifying as Hispanic/Latino, 52.7% identifying as White, 13% as Black/African American, and 5.6% as Asian. Of these respondents, 260 identified as women with faculty status. The survey data presented in this paper focus solely on these female faculty members.

In January 2018, 17 school and university leaders were invited to participate in the KI interviews. Informants were purposively sampled based on their background as faculty members in health sciences and/or their current position and its relevance to the subject matter being explored. Due to time constraints, 14 interviews were successfully completed. Participants included four University-level leaders, three of whom hold individual faculty appointments in one of the health units. Additional participants were executive-level leaders from the Schools of Medicine, Public Health, and Nursing, and the College of Arts and Sciences, which offers an undergraduate major in human health and houses several science departments.

The FCCS was considered institutional research and therefore exempt from ethical consideration by the Emory University Institutional Review Board (IRB). Emory's IRB also determined that the KI interviews were exempt from full review based upon the limited generalizability of the results. Nevertheless, procedural steps were taken to protect the rights of participants and ensure confidentiality throughout data collection, management, and analysis. Verbal informed consent was acquired from all participants before the KI interviews were conducted and participants were informed that they could withdraw from participation at any time. There was minimal risk to the participants, as their participation and the information collected from them were kept confidential.

### Data management and analysis

The survey was emailed to the staff and faculty by an external vendor. After data collection, the vendor provided Emory's Office of Institutional Research with a de-identified dataset. Descriptive analyses were performed in STATA to summarize faculty knowledge, attitudes, and experiences with sexual assault and harassment over their time at Emory.

All KI interviews were audio recorded and stored on a password-protected device. The interviewer took handwritten notes using symbols to identify salient points made in each interview. Interview notes were transcribed and coded using MAXQDA12 software (VERBI GmbH, Berlin, Germany). Deductive themes originated in the semi-structured interview guide; inductive themes were derived from the codes using grounded theory [24]. Saturation for all themes was reached after 11 interviews. Demonstrative quotes for each major theme were extracted verbatim from the audio recordings.

## Results

Two hundred and sixty women faculty participated in the FCCS, with 140 identifying their primary appointment in the professional schools comprising Health Sciences (Nursing, Medicine, Public Health, or Yerkes National Primate Center). Mean years of employment at Emory was 11.6 years (range 0–56 years, *n* = 250) with 33% of participants in tenure track positions and 24% with tenure. The majority of participants identified as White (85%), 5% Asian, 4% Black/African American, and 3% identified as Hispanic/Latina.

Among qualitative participants (*N* = 14), the average length of time at Emory was 18.8 years with a range of 7–38 years. All KIs identified as women; three were women of color.

**Fig. 1.**
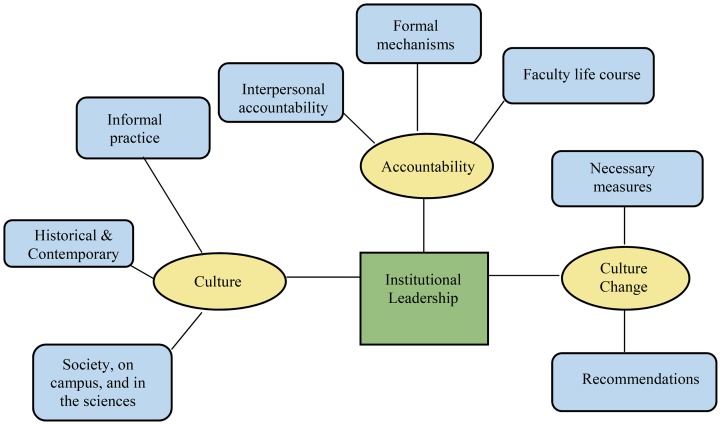
Thematic network.

Three core organizational themes emerged from the data: culture, accountability, and culture change. Thematic network analysis was used to map the relationships between themes (see Fig. 1) [25]. A global theme of institutional leadership was the unifying thread across these themes. Quantitative data from the FCCS relevant to each theme is presented followed by qualitative data.

### Culture

Campus climate was considered as a proxy for culture when considering the quantitative data. Since coming to Emory, 41% of women faculty reported having experienced an inappropriate comment about their own or someone else's body, appearance, or attractiveness capturing a climate of harassment. Two-thirds (59%) of women faculty had witnessed sexual harassment and 28% experienced sexual harassment while at Emory. Among respondents reporting they had experienced sexual harassment since coming to Emory, <2% used Emory's formal procedures to report the incident.

When asked in the interviews about their perceptions of how incidents of sexual harassment and assault had been handled on campus in the past, the theme of culture – both on and off campus – clearly emerged.

…we live in a culture that is misogynistic. Let's just be honest. So how do you fix something at a university level which in some sense, when you think about American society and American culture, is actually a very small, microcosm right? (#4-SL)

Narrowing in on academic culture led one participant to a troubling comparison

‘universities are like the Catholic Church. They sweep things under the rug as long as they can, and when they can't do that anymore they send the problem faculty away with a good reference. Look at what is happening right now at Michigan State. This happens all the time’ (#2-SL).

Participants noted a historical culture of silence that preferred patience over action.

The perception is this is an old guys problem…We should just be patient and try to quarantine people, I have witnessed a kind of reproduction of some of these behaviors and attitudes in the next generation (#1-SL).

A lack of onsite child care, and cultural practices like holding faculty or scholarly meetings during evening hours or other times when women who are primary care givers are not typically available were noted as harmful and exclusionary. The disproportionate number of women in clinical research/non-tenure track positions in the fields of public health and medicine was also noted as an informal barrier to traditional pathways of academic leadership.

When discussing culture and the sciences, one participant observed, ‘in certain departments, actually I think it's worse in the sciences…Bro culture, bro culture is alive and well’ (#6-UL). Women in the sciences were perceived as being held to a higher standard, ‘You have to be twice as good and work twice as hard’ (#8-SL). Another observed that science is inherently collaborative, requiring interdependency and understandings of hierarchy. Yet, these same qualities were seen as, ‘embodying systems of patronage, favor, power dynamics that can be gendered, negatively gendered, and negatively experienced by women and very harmful’ (#1-SL).

Variability across academic units came out strongly.

It's getting frustrating for women in the medical school at Emory. The glass ceiling feels very real, very real. And you know, women might get asked, women leaders might get asked to lead the search committee but they are not being asked first, ‘is this a position you might be interested in applying for?’ (#9-SL)

Yet, these negative views were not universal. Others believed that some disciplines, particularly public health, were more progressive than other science fields. One participant highlighted the difference between public health and other science fields,

public health is so many miles ahead of sciences—I mean hard sciences. The laboratory environment, and med school, and medicine, and that environment I think the whole, —it is a much more progressive culture. We're much more aware and sensitive to these kinds of issues… you know, that is a culture of public health. We're full of idealists who are more progressive politically; we're more progressive on all fronts. And so, its not to say that we don't have issues, it's that I think the culture here is just miles better than either medicine or hard sciences. I think laboratory environments are very intense and problematic. Um, and that that is a place where a lot of negative things can happen, uh, for women. And then, I think, medicine is often filled with cowboys who just don't have a lot of respect for women. So, I think we are really fortunate to be in this environment relative to the others. I would never want to—to be in the other environments personally (#3-SL).

Informal practices in support of women's advancement included unstructured affinity groups (e.g. Women in Science at Emory) and explicit attempts to amplify women's voices in meetings, presence on committees, and in leadership (i.e. by nominating women for awards).

### Accountability

Accountability in the context of the FCCS was measured by knowledge of and experience with formal mechanisms to address harassment, assault, and gender discrimination. Since starting at Emory, 70.8% of women faculty reported having received training/education about sex discrimination, including sexual harassment/sexual violence. Seventy-five percent reported they knew what Title IX is and the rights it protects, yet only 33% knew who the Title IX coordinator was or how to contact them. When asked who they would contact if they needed to report sexual assault/harassment, 43% noted they would contact their supervisor, 43% said police, 36% human resources, 35% Title IX Coordinator, 18% said Faculty and Staff Assistance Program, and 10% said another professor.

Overall, when asked if they felt comfortable guiding a colleague through the disclosure process, 16% reported they would be very uncomfortable doing so, with only 24% stating they would be very comfortable doing so. Reasons contributing to discomfort with assisting a colleague with disclosure include 36% being unsure how to report issues, 31% difficulty of subject matter, 15% noting fear of misreporting, 13.5% fear of inaction by the University, 12% fear of loss of confidentiality, 10% were concerned about how they would be treated, and 10% feared retaliation.

Both a lack of and a need for accountability featured prominently in the interviews. Most often accountability was discussed in terms of interpersonal accountability between leadership and others in positions of relative power, ‘until Deans and Department chairs hold mentors and mentees accountable it's [gender equity] not gonna happen…we need accountability’ (#2-SL).

Accountability was also inclusive of formal mechanisms providing protections from and recourse against gender discrimination. These included the Title VII and IX federal protections and their respective mandatory onboarding trainings. While participants acknowledged Emory's actions toward compliance with federal regulations, there was a perceived gap between legal compliance, a bare minimum, and true accountability, namely by key stakeholders in positions of power. The importance of implicit bias training in countering these behaviors was noted frequently as important, albeit with some caveats (Table 1; Formal mechanisms).

**Table 1.** Subthemes of accountability and demonstrative quotes among 14 female faculty key informants

Accountability subtheme	Quote
*Formal mechanisms*	‘I do know Emory has made great strides in having beefed up, or maybe having created Office of Equity and Inclusion, and they do I think good work on compliance in some respects and that is definitely an improvement. The challenge is always in the weeds. People in positions of power have to do what they need to do when they know that someone is violating the policies’ (#6-UL).
	‘It's not about having best practices. It's about applying strategies and holding people accountable…But training doesn't really equate to moving the needle. So, we all say we'll do better, and we go back to our units and we have a search and we forget to do better. Or we do better, or we think we've done better because we've been able to check the box that we have a woman in the search and that we have a minority in the search. But to really do better, someone has to hold people accountable for that. To date we really haven't had a robust strategy for holding departments, department chairs, search committees we haven't had a strategy to hold them accountable. So you don't see the needle moving’ (#7-UL).
*Faculty life course*	
Search committee composition and pools	‘We have some good guidelines for how to create a diverse pool, including gender…we need put some real teeth into our expectations, that pools are diverse and if there are not diverse then searches won't go forward. I think there are things that we can do to implement our own expectations around hiring that would help to get more women in leadership or more women in science’ (#5-UL).
Hiring	‘Have to say I've never heard that mentioned about a man. About whether they have a spouse that would need accommodations in terms of employment. Again, I have never heard that raised about a man that was interviewing for job. I think I hear it every single time about a woman who is being recruited…You know stuff like that still very much happens on a pretty regular basis, here and lots of other places’ (#5-UL).
Tenure process	‘APT [Appointments, Promotions and Tenure Committee] values members in National Academy of Science, National Academy of Medicine, you look at the numbers and it is ridiculous how disproportionality male dominated it is. Maybe, either we should really be trying to figure out how to get women in or we should discount these organizations that are completely, good ole boys clubs…So that would be something that the APT-level that maybe we could at least discuss’ (#3-SL).
Promotion	‘When women become associate professors they start doing service, and they do more service than men. I think the heavy reliance on women, and particularly minority women, to do service for the university on committees, to do the kind of housekeeping of the university I think hampers their ability to get promoted…which means we end up with full professors who are largely male’ (#6-UL).
Gender bias in parenthood	‘They see it as someone having more time, and therefore they should have gotten more done. So the bar is raised for that extra year. And that's just crazy. It's not uncommon that you have to remind somebody that it wasn't an extra year’ (#5-UL).
	‘sabbatical under the guise of parental leave…fathers who aren't primary care givers get an extra year and outpace the productivity of their female counterparts’ (#1-SL).

Specific university policies affecting the faculty life course were also noted for their importance. Policies related to diversity on faculty search committees and within candidate pools were viewed positively (Table 1; Search committee composition and pools). Formal processes related to hiring, tenure, and promotion came up frequently as opportune areas for improvement in considerations of women's leadership in its varied and often gendered forms (Table 1; Hiring; Tenure Process; Promotion).

Parental leave, delay in tenure review following the birth or adoption of a child, emergency child care, and caregiver leave policies were all generally seen as positive; however, potential unintended consequences were important considerations (Table 1; Gender bias in parenthood). Numerous university programs were also touted for either professional development or direct support to advancing women's leadership – even if not solely directed at women. These included on- and off-campus programs such as school-level mentoring programs, the Woodruff Leadership Academy, the Academic Leadership Program, The OpEd Project, and the Executive Leaders in Academic Medicine program among others.

### Culture change

The need for culture and social norms change within the university space was the perceived way forward among participants. Education directed at senior school-level leadership (e.g. Deans, Department chairs) was notable.

Chairs have to be taught, educated how to interpret policies and how to talk about things like childbirth, children even let's say breastfeeding in a way that doesn't make it seem like it's a burden or problem in a career…I think this issue of seeing the female biological clock and biology as a kind of problem for career is part of the fundamental part of culture that we have to change (#6-UL).

Additionally, participants recommended better data collection around women's workplace experiences, transparency in faculty searches, the intentional mentoring of faculty, a need for ongoing conversations around diversity and power, and expectation setting for a culture of both diversity and excellence; two concepts which are mutually reinforcing rather than mutually exclusive. Others suggested the need to prioritize gender equity at the highest levels of leadership, setting specific diversity targets in recruitment and retention of female faculty, and ways to address the leaky pipeline.

At every level we need to be thinking about what is the basis of the leaky pipeline and how do we try to not have that happen. Are there ways to identify where people are stepping off the pipeline and at least provide support for staying on (#3-SL).

Making use of faculty skill sets in the production of knowledge, ‘about systems of power and applying that to our own environment’ (#1-SL), was a sentiment that was echoed numerous times.

What can we do within the university setting and what's our responsibility to the larger society? Part of the goal of any academic institution is to contribute to the common good…common good means helping to fix some of the larger societal ills around our misogynistic culture…I think our responsibility requires looking inward but also looking outward at what we can do to contribute to the common good and society. If we just look inward we're going miss the boat (#4-SL).

A key feature in the likelihood of meaningful culture change was the role of institutional leadership.

### Institutional leadership

Four of Emory's nine deans are women and the president is a female faculty member whose faculty appointment is in the health sciences.

The thing is Emory is actually doing pretty well in higher leadership and women, you know. So in many ways I am proud to say I work at Emory for our particular president and at an institution that is, has placed women in leadership roles. And I think that again, is because we have someone at the top who is intentional about doing that…I think you could say that Emory is a wonderful classic case of what leadership can do actually (#6-UL).

Yet despite having achieved gender equity at the highest levels, one participant described female faculty as being ‘*appalled*’ that there was not a female finalist during a recent high-level search in the health sciences and several commented that the number of women chairs in the School of Medicine is quite low. Nevertheless, institutional leadership was seen as a key to advancing the strategy and methods to achieve gender equity for female faculty.

Participants acknowledged the varying needs of academic units. However, they uniformly agreed that school-level inconsistency and discretion in upholding gender-related policies have resulted in vastly different experiences for women across campus. One participant noted, ‘it is time to start acting like a mature university’ (#8-SL).

Specifically, participants recommended that Deans and Department Chairs be required to develop a diversity plan with concrete goals and metrics for accountability. In this way, school-specific needs could be respected while ensuring more uniform implementation of best practice standards for gender diversity and equity. The hiring of chief diversity officers within academic units was viewed as a positive development. Accurate budgeting to account for parental leave as the standard practice was another strong recommendation. Greater transparency at the school-level in terms of compensation across faculty rank and gender, and policies for search committees and tenure review were desired.

One participant summed up the role of leadership succinctly,

I'm not optimistic that gender bias is ever gonna go away anytime soon. I'm not optimistic about that because I think it's a problem that will always exist. It's just how we deal with it that will matter. And not just how we deal with it at here at Emory but how institutions in general deal with inequities (#7-UL).

The FCCS was not mandatory, so the data reflects the experiences of those who opted to participate. To protect confidentiality, we did not capture any data on those who chose not to participate in the survey. As a result, we do not have a formal way to compare respondents to non-respondents. It is possible that respondents differed in some way from non-respondents. The qualitative interviews are limited in that they were conducted in the months following the #MeToo movement when there was heightened attention to issues of sexual harassment, and assault. These data were undoubtedly influenced by this parallel social phenomenon. While not generalizable, the qualitative findings of this study may be transferable to similar university settings.

## Discussion

We examined the perceptions of female health sciences faculty with regards to policies and practices for the prevention of and response to sexual harassment, assault, and other gender inequities. Two-thirds (59%) of our respondents reported witnessing sexual harassment and nearly one-third (28%) reported experiencing it underscoring how prevalent psychological forms of gender-based violence – like sexual harassment – are. Despite widespread training on existing policies and protections (70.8%), only 2% had ever used a formal reporting mechanism. Our qualitative data revealed a culture of silence with participants expressing a range of reasons for not disclosing their experiences, not dissimilar to other forms of gender-based violence. Linked to this, university response to sexual harassment was seen as driven solely by compliance requirements. Variance across academic departments and units either lessened or worsened perceived gender inequities with medical settings and laboratories seen as more difficult environments than that of public health. There was a clear demand for institutional-wide metrics for accountability and greater transparency.

Campus Climate Survey research is a nascent field. Our survey – among the first of its kind – was implemented in response to federal recommendations and designed to inform policy at the campus and federal level. These kinds of surveys make an important contribution to our understanding of campus sexual violence but they have limitations. Standardization of sexual violence measures and sharing results need to be implemented [26]; still, the effort to study campus sexual violence on each campus through quantitative and qualitative methods is an important aim. Discussing these data, the challenges and limitations in gathering these data, and the implications of these data are an important way to understand campus sexual violence on the frontlines of where it occurs – within campuses.

Few colleges and universities surveyed faculty and staff experiences of sexual harassment and campus climate; Emory's commitment to include the entire campus community is a strength. The downside, however, is that it is difficult to find analogous data to compare Emory's response rate, findings, etc. The response rate for our faculty/staff survey was 20%. A systematic review of Campus Climate Surveys among students found that the modal response rate was between 10% and 19% [26]. Our response rate was congruent with that of Campus Climate Surveys among students.

As expected, the percentage of respondents who have witnessed sexual harassment was higher than the percentage who directly experienced sexual harassment for a number of reasons. First, multiple people could report seeing the same incident, and second, it is more likely to witness sexual harassment than to be a target of it.

Our findings on the experiences and perceptions of female faculty working in the health sciences are also consistent with other research on misogyny in the STEM fields [27]. These similarities include experiences of harassment, squandering women's potential along the ‘leaky pipeline,’ and impediments to women's advancement in leadership [28]. Among our participants, the culture within the US national context, on the university campus and within the sciences, was seen as playing a major role in creating a permissive environment for informal practices which resulted in harm to women in their career development and lives. When female faculty are experiencing harassment and gender discrimination themselves, it is difficult to create a campus culture where female students are encouraged to thrive thereby perpetuating the cycle of inequity.

## Conclusions

As the training ground and future workplace of many female scientists, university settings hold the potential and responsibility for the professional development and advancement of trainees and faculty alike. Our data highlight the ways in which university culture mimics the larger societal culture; they also show the ways in which culture in higher educational settings is distinct – namely the processes of faculty recruitment, tenure, and promotion, all of which were mentioned as processes in need of improvement. While federal anti-discrimination protections were lauded, accountability among department, school, and university leaders was seen as equally important in ensuring gender protections. Specific metrics, for example, on the recruitment of female faculty and accountability for those metrics is one example of concrete action. Although not an explicit topic of our study, universities should explore how to protect and promote gender, including gender non-conformity, in a way that respects the intersectional nature of identity.

Transparency and accountability for sexual harassment cases are equally important. The university bears the burden of balancing the confidentiality of the accuser and the accused, while simultaneously guaranteeing campus feelings of justice and accountability. Data about most cases are not readily available, with only the most egregious or high profile gaining media and public scrutiny. When information is sealed and cases remain confidential gossip, rather than transparent, conversation is often the result. If universities are serious about addressing sexual harassment and other gender inequities, the issues must be measured, reported, and transparently discussed. At times, this may require delays pending legal actions. Following investigation and legal determinations, records should not be permanently sealed.

Proposed legislation for increased accountability among top university administrators is likely to spur conversation within higher education about what accountability for university leaders should entail [29]. Whether mandated by legislation or not, our case study strongly concludes that university leaders must undertake measures to support mechanisms for formal and interpersonal accountability among senior leaders in their institutions. In this way, the intentional commitment of institutional leadership to strengthening accountability can advance the dual goals of culture change, as well as facilitate the achievement of values-based diversity goals to ensure the advancement of women in leadership roles both within and beyond the institution.

## References

[1] Brown E (2018) California professor, writer of confidential Brett Kavanaugh letter, speaks out about her allegation of sexual assault. *The Washington Post* (Internet). 2018 Sept 16 (cited 2018 Oct 5). Available at https://www.washingtonpost.com/investigations/california-professor-writer-of-confidential-brett-kavanaugh-letter-speaks-out-about-her-allegation-of-sexual-assault/2018/09/16/46982194-b846-11e8-94eb-3bd52dfe917b_story.html?utm_term=.45f67d73c14b.

[2] Zillman C (2017) Me Too: How Alyssa Milano's two-word protest against sexual harassment went viral. *Fortune* (Internet). 2017 Oct 16 (cited 2018 Jan 24). Available at http://fortune.com/2017/10/16/me-too-facebook-alyssa-milano/.

[3] Kantor J and Twohey M (2017) Harvey Weinstein paid off sexual harassment accusers for decades. *NY Times* (Internet). 2017 Oct 5 (cited 2018 Jan 30). Available at https://www.nytimes.com/2017/10/05/us/harvey-weinstein-harassment-allegations.html.

[4] Abramson A and Cooney S (2017). These are all the politicians recently accused of sexual harassment. *Times* (Internet). 2017 Dec 8 (cited 2018 Jan 30). Available at http://time.com/5033751/sexual-harassment-politicians-roy-moore-al-franken/.

[5] Levenson E (2018) Larry Nassar sentenced to up to 175 years in prison for decades of sexual abuse. *CNN* (Internet). 2018 Jan 24 (cited 2018 Jan 30). Available at https://www.cnn.com/2018/01/24/us/larry-nassar-sentencing/index.html.

[6] Jagsi R (2018) Sexual harassment in medicine – #MeToo. New England Journal of Medicine 378, 209–211. 29236567 10.1056/NEJMp1715962

[7] Whitworth JA (2016) Women and global health: a personal view. Global Health, Epidemiology and Genomics 15 June (1), e10 10.1017/gheg.2016.6. PMC5870405 29868202

[8] Gluckman N, Read B, Mangan K and Quilantan B (2018) Sexual harassment and assault in higher ed: what's happened since Weinstein. The Chronicle of Higher Education (Internet). 2018 Nov 17 (cited 2018 Jan 18). Available at https://www.chronicle.com/article/Tracking-Higher-Ed-s-MeToo/241757.

[9] Richter AR (2018) First women leaders in Global Health conference comes to Stanford. Scope Stanford University School of Medicine blog (Internet). 2018 Oct 11 (cited 2018 Jan 24). Available at http://scopeblog.stanford.edu/2017/10/11/first-women-leaders-in-global-health-conference-comes-to-stanford/.

[10] Richter R (2017) Conference advocates for more female leadership in global health. Stanford School of Medicine News Center (Internet). 2017 Oct 18 (cited 2018 Jan 24). Available at http://med.stanford.edu/news/all-news/2017/10/conference-promotes-more-female-leadership-in-global-health.html.

[11] Sloan J and Fisher B (2011) Constructing the sexual victimization of college women on campus In The Dark Side of the Ivory Tower. New York: Cambridge University Press, pp. 81–110.

[12] Fedina L, Holmes JL and Backes BL (2018) Campus sexual assault: a systematic review of prevalence research from 2000 to 2015. Trauma Violence Abus 19(1): 76–93. 10.1177/1524838016631129 26906086

[13] World Health Organization (2017) Violence Against Women. Geneva, Switzerland: World Health Organization Available at http://www.who.int/en/news-room/fact-sheets/detail/violence-against-women.

[14] Equal Employment Opportunity Commission (2019) Title VII of the Civil Rights Act of 1964 (Pub. L. 88-352) U.S. (Internet) 2019 Feb 28 (cited 2019 Apr 17). Available at https://www.eeoc.gov/laws/statutes/titlevii.cfm.

[15] U.S. Department of Justice (2015) Overview of Title IX of The Education Amendments Of 1972, 20 U.S.C. A§ 1681 Et. Seq. (Internet). 2015 Aug 7 (cited 2018 Jan 22). Available at https://www.justice.gov/crt/overview-title-ix-education-amendments-1972-20-usc-1681-et-seq.

[16] National Archives and Records Administration (2014) Memorandum – establishing a White House Task Force to protect students from sexual assault. (Internet). 2014 Jan 22 (cited 2018 Jan 22). Available at https://obamawhitehouse.archives.gov/the-press-office/2014/01/22/memorandum-establishing-white-house-task-force-protect-students-sexual-a.

[17] White House Task Force to Protect Students from Sexual Assault (2014) Not alone: the first report of the White House Task Force to protect students from sexual assault (Internet). 2014 Apr (cited 2018 Jan 22). Available at https://www.justice.gov/ovw/page/file/905942/download.

[18] US Department of Justice Office on Violence Against Women (2017) National progress on campus climate surveys: a snapshot. 2017 Jan (cited 2018 Jan 22). Available at https://www.justice.gov/ovw/page/file/929841/download.

[19] Breiding MJ, Smith SG, Basile KC, Walters ML, Chen J and Merrick MT (2014) Prevalence and characteristics of sexual violence, stalking, and intimate partner violence victimization – national intimate partner and sexual violence survey, United States, 2011. MMWR 63, 1–18. PMC4692457 25188037

[20] Sales JS and Krause KH (2017) Schools must include faculty and staff in sexual violence prevention efforts. Journal of American College Health 65, 585–587. 28665189 10.1080/07448481.2017.1349133

[21] Emory University Woodruff Health Science Center (2017) US News and World Report: 2017 rankings (Internet) (cited 2018 Jan 22). Available at http://whsc.emory.edu/about/facts-and-figures/rankings.html.

[22] Emory University Woodruff Health Science Center (2018) Comprehensive figures (Internet) (cited 2018 Jan 22). Available at http://whsc.emory.edu/about/facts-and-figures/figures.html.

[23] Emory University Facts & Figures (2018) (Internet). (cited 2018 Jan 22). Available at http://www.emory.edu/home/about/factsfigures/index.html.

[24] Hennink MM, Hutter I and Bailey A (2011) Qualitative Research Methods. London: Sage.

[25] Attride-Stirling J (2001) Thematic networks: an analytic tool for qualitative research. Qualitative Research 1, 385–405.

[26] Krause KH, Woofter R, Haardoerfer R, Windle M, Sales JS and Yount KM (2018) Measuring campus sexual assault and culture: a systematic review of campus climate surveys. Psychology of Violence (1 October).

[27] Funk C and Parker K (2018) Women and men in STEM often at odds over workplace equity. Pew Research Center (Internet). 2018 Jan (cited 2018 Jan 22). 158 p. Available at http://assets.pewresearch.org/wp-content/uploads/sites/3/2018/01/09142305/PS_2018.01.09_STEM_FINAL.pdf.

[28] The Lancet (2018) Year of reckoning for women in science. Lancet 391, 513. 29617222 10.1016/S0140-6736(18)30238-1

[29] Harris A (2018) Bill would hold college presidents accountable for sexual abuse by employees. The Chronicle of Higher Education (Internet). 2018 Feb 15 (cited 2018 Feb 23). Available at https://www.chronicle.com/article/Bill-Would-Hold-College/242558?cid=at&utm_source=at&utm_medium=en&elqTrackId=7632bd0e43a44b3c8c1f5161a78d4bfa&elq=89e72ab0fb6c4eafa8a6df88cb4b88f7&elqaid=17872&elqat=1&elqCampaignId=7910.

